# Adverse events associated with use of immunoglobulin in pediatric patients reported to the US Food and Drug Administration Adverse Event Reporting System, 2001–2023

**DOI:** 10.1111/pai.70319

**Published:** 2026-03-10

**Authors:** Shaokui Wei, Yeowon Kim, Mary N. Rubin, Srinivas S. Ayyala, Kerry Welsh

**Affiliations:** ^1^ Office of Biostatistics and Pharmacovigilance, Center for Biologics Evaluation and Research Food and Drug Administration Silver Spring Maryland USA

**Keywords:** adverse events, FAERS, immunoglobulin, pediatrics, postmarketing

## Abstract

**Background:**

Immunoglobulin products are widely used for the treatment of immunodeficiency and autoimmune disorders. Although clinical trials have demonstrated their efficacy and tolerability, data describing their postmarketing safety profile in pediatric populations remain limited, particularly regarding rare and serious adverse events. This study aimed to characterize adverse event reports associated with immunoglobulin use in children during the postmarketing period.

**Methods:**

Pediatric adverse event reports (<18 years) submitted to the U.S. Food and Drug Administration Adverse Event Reporting System between January 1, 2001 and December 31, 2023 were analyzed. Descriptive statistics were used to summarize reported adverse events, and selected events and deaths were reviewed individually.

**Results:**

In total, 2997 pediatric adverse event reports were identified, of which 1855 (61.9%) were classified as serious. The most frequently reported adverse events were headache (*n* = 394), pyrexia (*n* = 294), urticaria (*n* = 231), vomiting (*n* = 229), nausea (*n* = 182), and infusion‐related reaction (*n* = 151). Clinically significant adverse events were infrequent, including hemolytic disorders (*n* = 82), aseptic meningitis (*n* = 80), anaphylaxis (*n* = 34), thromboembolic events (n = 34), renal impairment (*n* = 22), and transfusion‐related acute lung injury (*n* = 7). Adverse event patterns were similar between intravenous and subcutaneous formulations, except for higher proportions of systemic infusion reactions with intravenous administration and injection‐site reactions with subcutaneous administration. Among 110 reported deaths, the leading causes were unspecified (*n* = 37), nervous system disorders (*n* = 20), infections (*n* = 16), and general disorders (*n* = 11).

**Conclusion:**

The postmarketing safety profile of immunoglobulin in pediatric patients was consistent with clinical data observed in prelicensure studies and described in the package insert, with no new safety concerns identified. This assessment is subject to important limitations, including reliance on passive surveillance data and the lack of exposure denominators, which should be considered when interpreting the results.

**Key message**

This study assessed the postmarketing safety profile of immunoglobulin in pediatric patients. The US Food and Drug Administration's Adverse Event Reporting System received 2997 reports for immunoglobulin in pediatric patients from 2001 through 2023. The most frequently reported adverse events included headache, pyrexia, urticaria, vomiting, nausea, and infusion‐related reactions. Clinically significant adverse events, including aseptic meningitis, hemolytic disorders, anaphylactic reaction, thromboembolic events, renal impairment, and transfusion‐related acute lung injury, were rare in pediatric patients. Postmarketing adverse events for immunoglobulin in pediatric patients were consistent with the safety experience observed in prelicensure clinical trials and described in the package insert.

## 1 INTRODUCTION

Polyclonal human immunoglobulin (IG) has been utilized for decades in treating immunodeficiency diseases.^1^ Derived from pooled plasma of healthy blood donors, IG products contain at least 96% immunoglobulin G, with trace amounts of immunoglobulin A and other plasma proteins. There are currently more than a dozen FDA‐approved IG products,^2^ which generally share similar safety and efficacy profiles. These products are available in two formulations: immunoglobulin intravenous (IGIV) and immunoglobulin subcutaneous (IGSC). In the United States (US), approved indications for IG include primary immunodeficiency (PI), idiopathic thrombocytopenic purpura (ITP), Kawasaki disease (KD), and certain neurologic disorders, such as chronic inflammatory demyelinating polyneuropathy (CIDP),^3^ and many IG products are approved for use in pediatric patients. Additionally, IG products are frequently used off‐label for various neurologic, dermatologic, rheumatologic, and infectious diseases.^4^

Clinical trials have demonstrated that IG is effective and well tolerated for their approved indications; however, adverse events (AEs) have been reported both in clinical trials and with post‐approval commercial use.^5^, ^6^ The common and mild AEs include headache, fever, flushing, malaise, chills, fatigue, and lethargy. More clinically significant but less frequent AEs include anaphylactic reaction, thrombosis, renal impairment, aseptic meningitis, hemolytic anemia, and transfusion‐related acute lung injury.^7^ Strategies such as assessing patient risk factors, premedicating, infusing IG at a slow rate, and switching from IGIV to IGSC can help mitigate these AEs.

While the safety profile of IG is well characterized in adults, data on pediatric patients are less robust due to the small number of pediatric subjects enrolled in clinical trials. To address this information gap, we reviewed AEs associated with IG use in pediatric patients reported to the US FDA's Adverse Event Reporting System (FAERS) from 2001 to 2023.

## 2 METHODS

### 2.1 FAERS database

FAERS is a passive surveillance system that collects spontaneously reported AEs, medication errors, and product quality issues associated with drugs and biological products.^8^ It's important to note that anyone can submit reports to FAERS, including healthcare professionals, consumers, and manufacturers. The reports in FAERS are not necessarily causally related to the product in question, and the events reported may not have been medically confirmed in all cases. Reports include information on patient demographics, medical history, concomitant medications, AE description, outcomes, and report sources. Events are coded using the Medical Dictionary for Regulatory Activities (MedDRA), which includes broad (system organ class [SOC]) and specific event categories (e.g., preferred term [PT]).^9^ The adverse event (represented by the specified PT) is considered a labeled event if it is described in the US package insert (USPI) but may not indicate drug‐related adverse event. Our analysis utilized MedDRA version 27.0. Each FAERS report can include one or more PTs and corresponding primary (and secondary, if applicable) SOC(s). Reports can be submitted by product manufactures as expedited (i.e., serious and unexpected [unlabeled] AEs reported within 15 days), or non‐expedited (i.e., serious expected and nonserious events reported periodically). Reports can also be submitted directly by health care professionals or consumers (“direct reports”). A report is considered serious if the AE results in death, life‐threatening conditions, hospitalization, permanent disability, birth defect, or other.^10^ The outcome of the AEs is specified by the reporter, with multiple outcomes possible per report.

We searched reports submitted to FAERS from January 1, 2001, to December 31, 2023, with an IG listed a suspect product using “Human Immunoglobulin G” and “Human Immunoglobulin G\Hyaluronidase (Human Recombinant)” as product active ingredient, encompassing all IG products approved by the FDA (Table S1). The reports were limited to U.S. reports and pediatric patients (under 18 years). The reports that did not specify age were excluded from the study. Foreign reports were excluded due to potentially differing clinical practice and drug use patterns. Follow‐up information for these reports could be submitted until June 19, 2024, making the study's data lock point.

### 2.2 Data analysis

#### 2.2.1 Descriptive analysis

We assessed reports by seriousness, frequency, and by route of administration (IGIV versus IGSC). The frequency was analyzed using descriptive statistics (Stata/IC 13.1; StataCorp LLC), without considering potential duplicate reports.

#### 2.2.2 Case reviews

Based on FAERS search findings, we reviewed reports of selected clinically significant AEs, such as anaphylactic reaction, thromboembolic events (TEEs), aseptic meningitis, hemolytic disorders, renal impairment, and transfusion‐related acute lung injury (TRALI). Except for aseptic meningitis and TRALI, the reports for these events were identified through a standardized MedDRA Queries (SMQ) [Narrow] (Table S2). We also reviewed all death reports to determine causes of death, assigning causes when not explicitly stated. Time to death was calculated from the last known IG infusion date, using the infusion start date when the last infusion date was missing. Duplicate reports were excluded from individual case review when possible.

#### 2.2.3 Ethics

This safety review did not require institutional review board approval or informed patient consent, as it met the exemption criteria set by the Office for Protection from Research Risks under Department of Health and Human Services regulations.^11^

## 3 RESULTS

We identified a total of 2997 reports following IG administration, of which 1855 (61.9%) were serious (Table 1). The median (interquartile range [IQR]) patient age was 10 (5–14) years, with a higher proportion of reports for males (53.3%) and individuals aged 7 to <18 years (66.6%). Nearly half of the reports (49.2%) were expedited reports, and 40.0% involved subcutaneous administration. Among these reports, 110 reports indicated an outcome of death.

**TABLE 1:** Description of US Adverse Event Reports for Immunoglobulin in Pediatric Patients Submitted to FAERS January 1, 2001 to December 31, 2023.

Characteristic	All reports	Serious reports^a^
	No. (%)	No. (%)
Total reports	2997 (100)	1855 (100)
Sex		
Females	1313 (43.8)	838 (45.2)
Males	1598 (53.3)	964 (52.0)
Not Reported	86 (2.9)	53 (2.9)
Age (year)		
0 to <1 year	147 (4.9)	122 (6.6)
1 to <3 years	253 (8.4)	179 (9.6)
3 to <7 years	601 (20.1)	353 (19.0)
7 to <18 years	1996 (66.6)	1201 (64.7)
Received at FDA (calendar year)		
2001–2005	120 (4.0)	105 (5.7)
2006–2010	239 (8.0)	195 (10.5)
2011–2015	554 (18.5)	482 (26.0)
2016–2020	1334 (44.5)	642 (34.6)
2021–2023	750 (25.0)	431 (23.2)
Type of report		
Expedited (15‐day)	1476 (49.2)	1370 (73.9)
Not expedited	1072 (35.8)	166 (8.9)
Direct	449 (15.0)	319 (17.2)
Administration route^b^		
Intravenous	388 (12.9)	281 (15.1)
Subcutaneous	1200 (40.0)	528 (28.5)
Not specified	1427 (47.9)	1060 (57.1)
Reported outcome^c^		
Death	110 (3.7)	110 (5.9)
Hospitalized, disabled, life‐threatening, congenital anomaly, and required intervention	935 (32.2)	935 (50.4)
Other	1217 (41.9)	1217 (65.6)
Not applicable^d^	1142 (39.3)	N/A

^a^ Reports that meet criteria for seriousness as defined in the Code of Federal Regulations^10^ (i.e., death, hospitalization, life‐threatening, disability, congenital anomaly, required intervention, or other serious outcomes) are considered serious.

^b^ One or more products may be specified per report.

^c^ One or more outcomes may be specified per report.

^d^ Refers to nonserious reports.

### 3.1 Most frequently reported PTs

In total, 10,458 PTs were reported across the 2997 case reports. The most frequently reported PTs included headache (*n* = 394), pyrexia (*n* = 294), urticaria (*n* = 231), vomiting (*n* = 229), nausea (*n* = 182), off‐label use (*n* = 154), and infusion‐related reaction (*n* = 151) (Table 2). These PTs were also frequent among serious reports in addition to drug ineffective (*n* = 115).

**TABLE 2:** Top Preferred Terms (PTs) for US Adverse Event Reports for Immunoglobulin in Pediatric Patients Submitted to FAERS January 1, 2001 to December 31, 2023.

Preferred term (PT)^a^	All reports	Serious reports^b^	Comment^d^
	No. (%)^c^	No. (%)^c^	
Headache	394 (13.2)	227 (12.2)	1
Pyrexia	294 (9.8)	187 (10.1)	1
Urticaria	231 (7.7)	104 (5.6)	1
Vomiting	229 (7.6)	154 (8.3)	1
Nausea	182 (6.1)	129 (7.0)	1
Off label use	154 (5.1)	108 (5.8)	3
Infusion related reaction	151 (5.0)	107 (5.8)	1
Rash	144 (4.8)	70 (3.8)	1
Sinusitis	137 (4.6)	86 (4.6)	2
Pruritus	130 (4.3)	47 (2.5)	1
No adverse event	123 (4.1)	51 (2.7)	3
Drug ineffective	122 (4.1)	115 (6.2)	3
Fatigue	122 (4.1)	70 (3.8)	1
Infusion site pain	106 (3.5)	36 (1.9)	1
Dyspnea	98 (3.3)	83 (4.5)	1
Infusion site erythema	90 (3.0)	27 (1.5)	1
Meningitis aseptic	85 (2.8)	77 (4.2)	1
Cough	84 (2.8)	61 (3.3)	1
Pneumonia	84 (2.8)	84 (4.5)	1, 2
COVID‐19	78 (2.6)	72 (3.9)	2
Ear infection	77 (2.6)	44 (2.4)	2
Product dose omission issue	77 (2.6)	58 (3.1)	3
Infusion site swelling	76 (2.5)	19 (1.0)	1
Infusion site discharge	73 (2.4)	17 (0.9)	1
Nasopharyngitis	73 (2.4)	45 (2.4)	1, 2
Chills	69 (2.3)	50 (2.7)	1
Erythema	69 (2.3)	34 (1.8)	1
Seizure	68 (2.3)	66 (3.6)	1
Upper respiratory tract infection	67 (2.2)	41 (2.2)	1, 2
Pain	64 (2.1)	31 (1.7)	1
Infection	62 (2.1)	51 (2.7)	1, 2
Diarrhea	61 (2.0)	37 (2.0)	1
Malaise	61 (2.0)	38 (2.0)	1
Product use in unapproved indication	60 (2.0)	44 (2.4)	3

^a^ PTs describing adverse events that are included in the US Package Insert are considered labeled events; however, not all PTs represent adverse events. A PT included in a FAERS report does not imply causality.

^b^ Reports that meet criteria for seriousness as defined in the Code of Federal Regulations^10^ (i.e., death, hospitalization, life‐threatening, disability, congenital anomaly, required intervention, or other serious outcomes) are considered serious.

^c^ One report may contain more than one PT. The percent of cases containing an event is calculated by dividing the count for the event by the total number of cases, multiplied by 100.

^d^ 1 = PT is a labeled AE or related to a labeled AE in the US package insert for at least one IG product; 2 = PT is related to infection or confounded by the indication; 3 = PT is not an AE but is related to product use issues or product efficacy.

We further analyzed PTs by IG subgroups: immunoglobulin intravenous (IGIV) and immunoglobulin subcutaneous (IGSC). While there was considerable overlap in the most frequently reported PTs, systemic reactions, including infusion‐related reaction, chills, aseptic meningitis, dyspnea, hypotension, and anaphylactic reaction, were more prevalent with IGIV, whereas injection site reactions, including infusion site pain, infusion site erythema, infusion site swelling, and infusion site discharge, were more common with IGSC (Figure S1).

### 3.2 Case review

#### 3.2.1 Death reports

A total of 110 reports with an outcome of death were identified in FAERS during the study period (Table 3). The median [IQR] patient age was 9 [2–15] years. There were 72 reports for which the time interval from IG infusion to death was available. Of them, the majority of deaths (51 [71.8%]) occurred after 30 days of IG infusion with a median interval between IG infusion and death of 4 months (range, <1 day−28.6 years). Most death reports were attributed to unspecified (37 [33.6%]), nervous system disorders (20 [18.2%]), infections and infestations (16 [14.5%]), and general disorders (11 [10.0%]). For death reports with unspecified (unknown) cause, there was not enough information present to determine the cause after manually reviewing case.

**TABLE 3:** Characterization of US Death Reports for Immunoglobulin in Pediatric Patients Submitted to FAERS January 1, 2001 to December 31, 2023.

Characteristic	Death reports	
	No.	%
Total death reports	110	100.0
Sex		
Females	45	40.9
Males	53	48.2
Not specified	12	10.9
Age (year)		
<1 year	13	11.8
1 to <3 years	19	17.3
3 to <7 years	16	14.5
7 to <18 years	62	56.4
Indication^a^		
Primary immunodeficiency syndrome (PID)^b^	29	26.4
Off label use	20	18.2
Hypogammaglobulinaemia	16	14.5
Post‐transplant	10	9.1
Kawasaki's disease	8	7.3
Hematopoietic stem cell transplant (HSCT)	8	7.3
Unspecified immunodeficiency	7	6.4
Encephalitis	5	4.5
Immune thrombocytopenia	2	1.8
Not specified	9	8.2
Time from treatment to death (day)		
<1	4	3.6
1–<7	6	5.5
7–<30	11	10.0
>30	51	46.4
Not specified	38	34.5
Causes of death		
Nervous system disorders	20	18.2
Infections and infestations	16	14.5
General disorders	11	10.0
Vascular disorders	9	8.2
Neoplasm	7	6.4
Respiratory disorders	7	6.4
Immune system disorders	2	1.8
Cardiac disorders	1	0.9
Not specified (unknown causes)^c^	37	33.6

^a^ One or more indications may be specified per report.

^b^ PID includes immunodeficiency common variable, X‐linked hyper‐IgM syndrome, severe combined immunodeficiency, and selective deficiency immunoglobulin G subclasses.

^c^ After manually reviewing, there was not enough information present to determine the cause.

Four deaths occurred within one day of IG infusion: three involved heart transplant patients who died during the infusion from unknown causes, and one involved a patient with vesicostomy and klebsiella peritonitis who died nine hours post‐infusion, potentially from acute myocardial ischemia. Six deaths were reported within one to seven days post‐infusion: four were attributed to underlying diseases (two from Kawasaki disease, one from disseminated adenoviral infection, one from acute hemorrhagic leukoencephalitis), one was due to bilateral internal jugular vein thrombosis in a patient with ITP, and one had an unknown cause. Among the 11 deaths reported within 7 to 30 days post‐infusion, seven were due to underlying diseases (two cases of hypogammaglobulinemia with infection, one each for Kawasaki, hemophagocytic lymphohistiocytosis, macrophage activation syndrome, acute lymphocytic leukemia with neuroinvasive West Nile virus, and bone marrow transplant with pulmonary graft‐versus‐host disease), while four deaths had unknown causes.

#### 3.2.2 Clinically significant AEs

We reviewed reports of selected clinically significant AEs: aseptic meningitis, anaphylactic reaction, thromboembolic events, hemolytic disorders, renal impairment, and transfusion‐related acute lung injury (Table 4).

**TABLE 4:** Characterization of Clinically Significant Adverse Event Reports for Immunoglobulin in Pediatric Patients Submitted to FAERS January 1, 2001 to December 31, 2023.

Adverse events	Number of reports *N* (%)	Age (year)^a^	Sex (female/male)	Time to onset^b^	Outcome (death) *N*
Hemolytic disorders	82 (2.7)	4.0	39/40	Within 7 days	2
Aseptic meningitis	80 (2.7)	11.0	45/35	Within 2 days	0
Anaphylactic reaction	34 (1.1)	8.0	12/22	During infusion	0
Thromboembolic events	34 (1.1)	13.0	17/17	Within 7 days	4
Renal impairment	22 (0.7)	12.5	6/15	Within 1 day	5
Transfusion‐related acute lung injury	7 (0.2)	11.0	3/4	Within 6 hours	1

^a^ Median age.

^b^ Of reports with time to onset information available, more than 50% of reports occurred in this timeframe.

##### Aseptic meningitis

Eighty unique reports of aseptic meningitis were identified, with 73 (91.3%) being serious. The median [IQR] age of patients was 11.0 [8.0–14.0] years, predominantly females (45 [56.3%]) and those aged 7 to <18 years (62 [77.5%]). Among the 52 reports indicating event onset, 44 (84.6%) occurred within 48 hours post‐infusion. All 45 patients with recovery status reported recovery within 1–2 days after onset, with no fatal outcome recorded.

##### Anaphylactic reaction

We identified 34 cases of anaphylactic reaction, all serious, including six cases of anaphylactic shock. The median [IQR] age of patients was 8.0 [4.7–15.0] years, with a majority of reports for males (22 [64.7%]) and individuals aged 7 to <18 years (21 [61.8%]). All 19 reports with event onset indicated that reactions occurred during IG infusion. All events were resolved following appropriate treatment (e.g., epinephrine at the infusion center, emergency department, or home; other treatments such as corticosteroids and antihistamine), with no fatalities reported.

##### Thromboembolic events

We identified 34 unique reports of thromboembolic events (TEEs), all serious, including four deaths. The median [IQR] age of patients was 13.0 [8.0–15.0] years, with an equal number of males and females (17 each). Of the 16 reports with assessable onset timing, 11 (68.8%) experienced events within 7 days of the last IG infusion. Nineteen reports (55.9%) cited at least one risk factor for TEEs, such as recent surgery, malignancy, systemic lupus erythematosus, vasculitis, estrogen‐containing hormonal therapy, or another potential cause of TEEs. Among the four deaths, three were likely caused by TEEs following IG administration and occurred less than 1 week following IG; two patients had pre‐existing risk factors for TEEs.

##### Renal impairment

Twenty‐two unique reports of renal impairment were identified, all serious, with five deaths. The median age was 12.5 years (range, 0.0–17.9 years). There were 15 males (68.2%) and 6 females (27.3%). The median onset for the nine reports with timing data was 1 day (range, 1–60 days). Five patients (22.7%) had pre‐existing renal conditions, including two who underwent renal transplants prior to IG therapy. Twelve patients (54.5%) had alternate diagnoses, such as nephrotoxic therapies or multi‐organ failure. All deceased patients had significant comorbidities, with no deaths directly attributed to renal impairment.

##### Hemolytic disorders

We identified 82 unique reports of hemolytic disorders, with 77 (93.9%) being serious, including two deaths. The median [IQR] age of patients was 4.0 [2.0–8.0] years, with 40 males (48.8%) and 39 females (47.6%). Kawasaki disease was the primary indication for IG use in 47 cases (57.3%). Seventy‐three of the 82 reports (89.0%) were consistent with hemolysis following IG administration. Of 51 reports with time to onset information available, 42 (82.4%) experienced hemolysis within 7 days, predominantly 2–3 days post IG infusion. One notable death case involved a 3‐month‐old female patient with suspected Kawasaki disease who developed IG‐related hemolytic anemia after multiple transfusions and died 15 days after IGIV infusion due to multisystem organ failure and possible hemophagocytic lymphohistocytosis.

##### Transfusion‐related acute lung injury (TRALI)

Seven unique reports of TRALI were identified, all serious, including one death. The median age was 11.0 years (range, 0.3–15.6 years). There were four males and three females. The most common indication for IG use were primary immunodeficiency (2 [28.6%]) and idiopathic thrombocytopenic purpura (2 [28.6%]). Four patients experienced symptoms within 6 hours of IG administration, which is a required criteria for a diagnosis of TRALI, but only two of these patients had symptoms consistent with TRALI. The remaining reports were either unclassifiable or related to other lung pathologies. The fatal case involved a patient with multiple comorbidities, with an indeterminate cause of death.

#### 3.2.3 Other AEs

We also reviewed additional AEs, including hepatitis B core antibody positivity, seizure, musculoskeletal stiffness, coronary artery dilation, and opsoclonus–myoclonus. The finding of hepatitis B core antibody positivity was attributed to passive transfer of hepatitis B antibodies.^12^ The AEs of seizure and musculoskeletal stiffness were manifestations associated with aseptic meningitis. Coronary artery dilation and opsoclonus–myoclonus were considered confounded by their underlying indications of Kawasaki disease and opsoclonus–myoclonus syndrome, respectively.

## 4 DISCUSSION

This study evaluated pediatric FAERS reports of AEs associated with IG administration over the past two decades, making it the largest postmarketing safety assessment of IG in pediatric patients to date. Our findings indicate that the reported AEs were consistent with the safety profile established in prelicensure clinical trials and described in the USPI.

Since its introduction in the early 1980s, the use of IG in the US and globally has progressively increased, particularly in recent years. IG therapy is a cornerstone for pediatric patients with PI, typically diagnosed at a young age, and is also used for conditions such as ITP, KD, CIDP, and hemolytic disease of the newborn due to ABO and Rh incompatibility. Balch et al.^13^ reported approximately 72,852 hospital admissions involving IG treatment for 53,648 unique patients across 47 US pediatric hospitals from 2007 to 2014, with AEs occurring in about 2%–25% of patients.

In our analysis, flu‐like symptoms (e.g., headache, fever, chills, nausea, vomiting, and fatigue), injection site reactions, and events related to the patient's underlying disease predominated among reported AEs. Systemic infusion reactions were more prevalent with IGIV, while injection site reactions were more common with IGSC. The findings align with previous studies showing that systemic AEs are less frequent with IGSC due to slower absorption,^14^ but mild to moderate injection site reactions are more frequent with IGSC.^15^ The differing safety profiles may stem not only from varying absorption rates but also from distinct indications for use. Balch et al.^13^ reported that at 47 US‐based children's hospitals from 2007 to 2014, PI accounted for only 1.2% of IGIV use, with KD and ITP being the primary indications, alongside Guillain‐Barré syndrome and prophylaxis of infections in patients undergoing antineoplastic chemotherapy. Typically, IGIV was administered in multiple consecutive doses and at a higher total dose for autoimmune or inflammatory conditions, while IGSC was primarily used for PI at a lower dose on a weekly schedule.

Clinically significant AEs, including anaphylactic reactions, thrombosis, aseptic meningitis, renal impairment, hemolytic anemia, and TRALI, have been well documented in adult populations. Most of these events, except for aseptic meningitis, were reported infrequently in pediatric patients. Anaphylactic reactions, often observed in plasma product transfusions, may be related to individual patient factors or donor‐related issues like trace amounts of specific antibodies, although in some cases the specific cause is never fully elucidated. We identified 34 cases of anaphylactic reaction, five of which were associated with IG lots that had been voluntarily withdrawn due to increased hypersensitivity reactions.^16^ TEEs are labeled events for IG use that have been reported to be associated with elevated plasma viscosity and procoagulant factor activation.^17^, ^18^ Several studies have shown that patients with ITP are at increased risk for thrombosis when receiving IGIV.^19^, ^20^ In this study, we identified 34 TEE cases, most of which occurred in patients with at least one risk factor for thrombosis. Aseptic meningitis is a serious neurological disorder following IG infusion. We identified 80 cases of aseptic meningitis, making it one of the most frequently reported AEs, especially in patients receiving IGIV. Other neurological disorders, such as headache and seizure, were also prevalent among the frequently reported AEs. While it remains unclear if these neurological manifestations share a common underlying mechanism or if pediatric patients are more susceptible, the frequently reported neurological events may be related to a higher dose of IG administered and varied indications for use in pediatric patients. Prior to 2000, the IGIV‐associated acute renal injury was a significant safety concern. In November 1998, the FDA issued an “Important Drug Warning”, noting over 114 reports globally (83 from the US) of acute renal dysfunction, including 17 fatalities, following the administration of various manufacturers' IG products.^21^ Epstein et al.^22^ reported that sucrose‐containing IG preparations might be associated with a risk of osmotic nephritis; however, most currently available IG products do not contain sucrose. In this study, we identified 22 cases of renal impairment, with many of these patients having pre‐existing renal diseases or other comorbidities. Hemolysis commonly occurs with high dose IG derived from non‐group O blood and is associated with the presence of A and B isoagglutinins (anti‐A and anti‐B antibodies) in IG products.^23^, ^24^, ^25^ A systematic review by Desborough et al.^25^ found that of 62 hemolysis cases, 97% occurred in patients receiving a high dose of IG (≥2 g/kg), and the majority of these patients were blood type A (65%) or AB (26%). In this study, we identified 82 cases of hemolytic disorders, primarily linked to high dose of IG use for Kawasaki disease, consistent with existing literature. A recent cohort study reported that excluding donors with high plasma titers of anti‐A antibodies and/or implementing immunoaffinity chromatography to reduce isoagglutinins A and B could lower the risk of hemolysis.^26^ TRALI was identified in only seven cases in our study, indicating that it is a rare occurrence following IG administration in pediatric patients.

Our analysis included 110 reports of death, with most attributed to unknown causes or underlying diseases. Four deaths were potentially related to TEEs (two from myocardial ischemia and two from stroke), and all of them involved patients with multiple risk factors for TEE, including ITP and KD. Additionally, two out of three deaths in heart transplant patients during IG infusion were documented in a single article,^27^ where the author speculated that increased plasma viscosity in context of graft coronary artery disease may have contributed to myocardial ischemia. Thus, heightened caution is warranted for patients with thrombotic risk receiving high doses of IGIV.

A significant strength of this study is its national representation of AEs associated with IG in a real‐world context over a postmarketing period exceeding 20 years. Although the data are limited to reports from the United States, the large size and long‐term nature of the dataset support the generalizability of the findings. Limitations include reliance on passive surveillance, which can lead to underreporting, duplicate reporting, varying report quality, and reporting bias (e.g., tendency for reporting more severe AEs, such as death, and those occurring closer to the time of product administration).^28^ We lacked information on product distribution and use, thereby limiting the assessment of AE frequency and reporting rates. Although our analysis cannot account for drug utilization, existing utilization data (e.g., Balch et al.^13^) provide context for interpreting reporting proportions. Additionally, we could not analyze AEs for individual IG product due to the aggregation of all suspect IG products into a single group, and the severity of AEs could not be evaluated in this study.

## 5 CONCLUSIONS

In conclusion, our analysis of FAERS reports did not reveal new safety concerns regarding IG use in pediatric patients. Most reported AEs were labeled events included in the USPI or confounded by the indications for product use. The emergence of new IG products and increased off‐label use in pediatric patients highlight the need for ongoing safety characterization of IGs, particularly for those with underlying medical conditions or at heightened risk for adverse events.

## AUTHOR CONTRIBUTIONS

**Shaokui Wei:** Conceptualization; writing – original draft; writing – review and editing; formal analysis; investigation; methodology. **Yeowon Kim:** Conceptualization; investigation; writing – review and editing; formal analysis; methodology. **Mary N. Rubin:** Conceptualization; investigation; writing – review and editing; formal analysis; methodology. **Srinivas S. Ayyala:** Conceptualization; writing – review and editing; investigation; formal analysis; methodology. **Kerry Welsh:** Conceptualization; supervision; investigation; writing – review and editing; formal analysis; methodology.

## CONFLICT OF INTEREST STATEMENT

The authors do not have any conflicts of interest. The opinions and information in this article are those of the authors and do not represent the views or policies of the U.S. Food and Drug Administration.

## Supporting information


Appendix S1.
