# Supplementary material for: Adverse events associated with use of immunoglobulin in pediatric patients reported to the US Food and Drug Administration Adverse Event Reporting System, 2001–2023
Source: Pediatr Allergy Immunol. 2026 Mar 10;37(3):e70319. doi: 10.1111/pai.70319 (PMC12975703; doi:10.1111/pai.70319)
Supplement: Supplementary file 1 — Appendix S1. [file PAI-37-e70319-s001.docx]

| **Supplement**  Figure 1    Figure 1. Comparison of top preferred terms (PTs) profile between intravenous immunoglobulin (IGIV) and subcutaneous immunoglobulin (IGSC) in pediatric patients. The heatmap displays the percentage of reports for most frequently reported PTs associated with IGIV and IGSC, as reported in the FDA Adverse Event Reporting System (FAERS) from January 2001 through December 2023. Rows represent the route of immunoglobulin administration (IGIV vs IGSC), and columns represent individual PT. Cell color intensity corresponds to the proportion (%) of reports for each PT, with warmer colors indicating higher reporting percentages and cooler colors indicating lower percentages (scale shown in the color bar). Numeric values within each cell denote the exact percentage of reports. |
| --- |

**Table 1. Top Product Names of Immunoglobulin with US Adverse Events Reports in Pediatric Patients Submitted to FAERS, January 1, 2001, to December 31, 2023**

| **Product Name** | **Number of Reports (%)** |
| --- | --- |
| HIZENTRA | 1,022 (34.1) |
| HUMAN IMMUNOGLOBULIN G | 661 (22.1) |
| GAMUNEX-C | 335 (11.2) |
| GAMMAGARD LIQUID | 317 (10.6) |
| PRIVIGEN | 191 (6.4) |
| CUVITRU | 93 (3.1) |
| GAMMAGARD | 86 (2.9) |
| GAMMAGARD S/D | 54 (1.8) |
| VIVAGLOBIN | 39 (1.3) |
| GAMUNEX | 31 (1.0) |
| HYQVIA | 24 (0.8) |
| CARIMUNE | 21 (0.7) |
| OCTAGAM IMMUNE GLOBULIN (HUMAN) | 19 (0.6) |
| GAMMAPLEX | 18 (0.6) |
| IVEEGAM | 18 (0.6) |
| GAMIMUNE N | 15 (0.5) |
| HUMAN IMMUNOGLOBULIN G\HYALURONIDASE RECOMBINANT HUMAN | 14 (0.5) |
| CARIMUNE NF | 11 (0.4) |
| GAMMAR | 11 (0.4) |
| IVIGLOB EX | 11 (0.4) |
| XEMBIFY | 11 (0.4) |

**Table 2. Selected Clinically Significant Adverse Events Reports Associated with Immunoglobulin in Pediatric Patients Submitted to FAERS, January 1, 2001, to December 31, 2023**

| **Selected Clinically Significant Adverse Events** | **Search Terms Used (Preferred Term (PT)**  **or Standard MedDRA Queries (SMQ))** | **Number of Reports (deduplicated)** |
| --- | --- | --- |
| Hemolytic disorder | Haemolytic disorders (SMQ) [Narrow] | 104 (82) |
| Aseptic meningitis | Meningitis aseptic (PT) | 85 (80) |
| Thromboembolic events | Embolic and thrombotic events (SMQ) [Narrow] | 62 (34) |
| Anaphylactic reaction | Anaphylactic reaction (SMQ) [Narrow] | 38 (34) |
| Renal impairment | Acute renal failure (SMQ) [Narrow] | 28 (22) |
| Transfusion-related acute lung injury | Transfusion-related acute lung injury (PT) | 9 (7) |
